# The role of efferocytosis in inflammatory bowel disease

**DOI:** 10.3389/fimmu.2025.1524058

**Published:** 2025-02-18

**Authors:** Yi-Qian Liu, Zhan-Zhan Li, Yong-Li Han, Qing-Bo Wang

**Affiliations:** ^1^ Institute of Acupuncture and Moxibustion, Henan University of Chinese Medicine, Zhengzhou, Henan, China; ^2^ Academy of Traditional Chinese Medicine, Henan University of Chinese Medicine, Zhengzhou, China; ^3^ Acupuncture Department, The First Affiliated Hospital of Henan University of Chinese Medicine, Zhengzhou, Henan, China

**Keywords:** efferocytosis, inflammatory bowel disease, macrophages, intestinal mucosal repair, inflammatory response

## Abstract

Efferocytosis is the process by which various phagocytes clear apoptotic cells. In recent years, an increasing body of evidence has emphasized the importance of efferocytosis in maintaining internal homeostasis. Intestinal macrophages play a crucial role in modulating intestinal inflammation and promoting tissue repair. Inflammatory bowel disease (IBD) is a chronic, progressive, and relapsing condition, primarily marked by the presence of ulcers in the digestive tract. The exact mechanisms underlying IBD are not yet fully understood, and current treatment approaches mainly aim at repairing the damaged intestinal mucosa and reducing inflammatory responses to ease symptoms.This article provides new perspectives on IBD treatment and clinical management by examining the expression of macrophage efferocytosis-related molecules, the effects of efferocytosis on IBD development, the various roles of macrophage efferocytosis in IBD, and treatment strategies for IBD that focus on efferocytosis.

## 1 Introduction

Efferocytosis refers to the process by which apoptotic cells are cleared by various phagocytes. Increasing evidence over recent years has emphasized its crucial role in maintaining internal homeostasis. Billions of cells undergo apoptosis daily in the human body, and any delay in their clearance can result in secondary necrosis, leading to the release of toxic intracellular contents. This can, in turn, trigger pathological inflammation and autoimmune responses (1). As a result, defects in efferocytosis are strongly associated with the development of several inflammatory and autoimmune disorders. Inflammatory bowel disease (IBD) is a chronic, progressive, and relapsing condition, characterized primarily by ulcers in the digestive tract. It includes ulcerative colitis (UC) and Crohn’s disease (CD), which together affect 6 to 8 million people worldwide, significantly impacting their quality of life and daily activities, with an increasing incidence in recent years (2). Although the pathogenesis of IBD is associated with various factors, such as environmental exposure, genetic predisposition, gut microbiota imbalances, and immune system defects, the exact mechanisms are still not fully understood. Current treatment strategies focus primarily on repairing damaged intestinal mucosa and reducing inflammation to alleviate symptoms (3). This article reviews the expression of macrophage efferocytosis-related molecules, the impact of efferocytosis on IBD, and the multifaceted roles of macrophage efferocytosis in the disease.

## 2 The efferocytosis process

Macrophage efferocytosis differs from classical phagocytosis and requires the coordinated expression of multiple molecules to complete the process. It is generally divided into four stages: apoptotic cells release signals that attract macrophages, receptors on macrophages bind to these signals, macrophages then engulf the apoptotic cells, and the fusion of lysosomes and phagosomes leads to the digestion and degradation of the apoptotic cells and their contents (4).

### 2.1 Apoptotic cells emit “find me” signals and “eat me” signals

The “find-me” signals emitted by apoptotic cells are primarily soluble molecules, which can be classified into three categories: nucleotides, membrane lipids, and chemokines. Nucleotides, such as adenosine triphosphate (ATP) and uridine triphosphate (UTP), are the most prominent “find-me” signals for apoptotic cells (5), and their release is regulated by the plasma membrane protein Pannexin-1 (PANX1) (6, 7). Membrane lipids, which serve as specific “find-me” signals for apoptotic cells, include lysophosphatidylcholine (LPC) and sphingosine-1-phosphate (S1P). The activation of LPC is controlled by caspase-3-activated phospholipase A2, which converts phosphatidylcholine to LPC (8). On the other hand, sphingosine kinase 1 (SphK1) is upregulated in apoptotic cells, driving the secretion of S1P (9). Chemokines, such as Fractalkine (CX3CL1), are membrane-bound proteins, and studies suggest that the release of CX3CL1 can enhance the chemotaxis of macrophages (10). These soluble signals are released by apoptotic cells into the surrounding environment, guiding macrophages to accumulate at the target site and stimulating their capacity to clear apoptotic cells. Phosphatidylserine (PS) is the most extensively studied “eat-me” signal, although other signals include calreticulin (Crt) (11), pentraxin 3 (PTX3) (12), and changes in surface protein glycosylation or surface charge (13). CD47 (11, 14) is the most well-known “don’t-eat-me” signal (15), and together, these signals help distinguish dying cells from adjacent healthy cells, enabling macrophages to effectively phagocytose apoptotic cells (16, 17).

In addition, research from the Ravichandran group has identified a novel family of solute carriers (SLC) that are specifically modified during efferocytosis, which plays a significant role in regulating the uptake of apoptotic cells. SLC2A1 supports the phagocytosis of apoptotic cells in vitro and in vivo, not only promoting the initial engulfment of the first apoptotic cell but also facilitating the continued uptake of additional apoptotic cells (18). Furthermore, the release of lactate via SLC16A1contributes to promoting an anti-inflammatory environment.

### 2.2 Corresponding recognition receptors on the surface of macrophages

Phosphatidylserine (PS) is the most prominent “eat-me” signal found on apoptotic cells, capable of binding to macrophage surface receptors either directly or indirectly. In the direct method, PS binds directly to receptors such as T cell immunoglobulin mucin (TIM)–1, TIM-4 (19), brain-specific angiogenesis inhibitor 1 (BAI1) (20), stabilin-2 (21), and advanced glycation end product receptors (17). In the indirect method, PS binds to macrophage receptors via bridging molecules, including receptor tyrosine kinases Tyro-3, Axl, and Mertk (TAM), and integrins (22). These bridging molecules facilitate the connection between PS and macrophage receptors, including milk fat globule-EGF factor 8 protein (MFG-E8 or lactadherin) (23), growth arrest-specific factor 6 (Gas6) (24), and annexin 1 (ANXA1) (25), among others (26).

Furthermore, nucleotides such as ATP and UTP bind to the purinergic receptor P2Y2 on macrophage surfaces, while LPC and S1P bind to G protein-coupled receptors G2A and S1PR on macrophages, respectively (17). These interactions activate the macrophage efferocytosis receptors, which, through different signaling pathways, trigger cytoskeletal reorganization and enable the phagocytosis and clearance of apoptotic cells (26, 27).

### 2.3 Macrophage phagocytosis of apoptotic cells

When macrophage surface receptors bind to signals emitted by apoptotic cells, they activate the programmed clearance system within the macrophages. This process is initiated through the activation of key regulatory factors from the Rho family of small GTPases, such as Rac (28), via two primary mechanisms: the LDL receptor-related protein 1 (LRP1/CD91) (29) and the adaptor protein GULP (the mammalian equivalent of C. elegans ced-6) (30), or through the guanine nucleotide exchange factor (GEF) DOCK180 and the cell movement protein (ELMO) (20, 31). Activated Rac triggers actin remodeling through the WASP pathway, promoting actin polymerization to engulf or capture apoptotic cells, leading to membrane invagination and the internalization of the cells, which forms a phagosome. In addition to these pathways, a third potential signaling pathway may involve the tyrosine kinase Abl (ABL-1) inhibiting the Abl interactor AbI (ABI-1), which seems to counteract macrophage efferocytosis, though the precise mechanism remains unclear (32).

### 2.4 Degradation and digestion of apoptotic cells by phagosomes and lysosomes

After the engulfment of apoptotic cells, the large GTPase Dynamin-Vps34 (a phosphatidylinositol (3)-kinase) pathway is activated, which in turn activates the small GTPase Rab5, promoting lysosome maturation (33). Following this, Mon1a (C. elegans SAND-1) and its partner Ccz1 recruit Rab5 and Rab7 to the phagosome, activating Rab7 and facilitating the fusion of phagosomes with lysosomes (34, 18). Concurrently, acid hydrolases and nucleases are activated to degrade and acidify the engulfed apoptotic cell remnants (35, 36). **Table 1** presents the molecules involved in efferocytosis.

**Table 1:** presents the molecules involved in efferocytosis.

Efferocytosis-Related Processes	Surface ligands of apoptotic cells	Macrophage cell-surface receptors
Apoptotic cells release "find me" signals	ATP,UTP,LPC,S1P, CX3CL1	S1PR,P2Y,G2A,CX3CR1, CXCL
Apoptotic cells release "eat me" signals	PS,Crt,PTX3.et	Tim-1,Tim-4,Tyro-3,Axl,Mer, integrin,P2Y2,G2A,S1P1-5
Phagocytic process	–	Rac1,Rho,Rab5/7,LRP1,DOCK180, ABL-1
Degradation and digestion process	–	PPARs,LXR,ABCA1,Rab17, LC3

### 2.5 The outcome of efferocytosis

**Figure 1:**
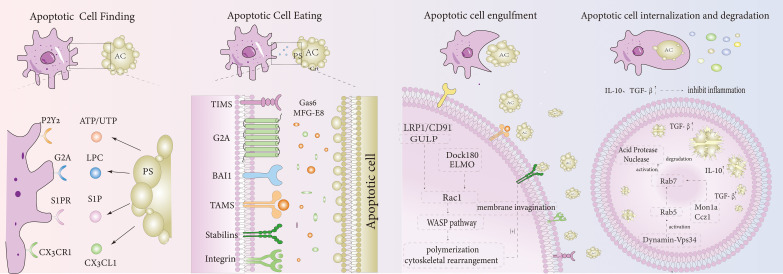
Efferocytosis mechanism diagram. Apoptotic Cell Finding: Apoptotic cells release recognition signals such as ATP/UTP, LPC, S1P, and CX3CL1, which bind to receptors on the macrophage surface, including P2Y2, G2A, S1PR, and CX3CR1, respectively. Apoptotic Cell Eating: Apoptotic cells express “eat me” signals like PS, which can bind directly to macrophage receptors such as TIMS, BAI1, and Stabilins. Additionally, PS can also bind to macrophage receptors indirectly through bridging molecules such as Gas6 and MFG-E8, which connect to TAMS and integrins. Apoptotic Cell Engulfment: After binding, apoptotic cells activate Rac1 through two pathways: LRP1/CD91/GULP and Dock180/ELMO. This activation triggers actin polymerization via the WASP pathway, leading to the formation of a phagocytic cup and the invagination of the plasma membrane. Apoptotic Cell Internalization and Degradation: After internalization, apoptotic cells are processed through the large GTPase Dynamin-Vps34 pathway, which activates Rab5 and promotes lysosomal maturation. Mon1a and Ccz1 subsequently recruit Rab5 and activate Rab7 on the phagosome. This Rab7 activation leads to the activation of acid hydrolases and nucleases, which degrade and acidify the engulfed apoptotic cell remnants. Anti- inflammatory mediators, such as TGF-β and IL-10, are released, dampening the inflammatory response.

The process of efferocytosis not only acts as a waste disposal mechanism to remove apoptotic cells but also leads to various biological outcomes. It can drive macrophage polarization toward a pro-resolution phenotype, release pro-resolving factors and anti-inflammatory mediators, promote self-tolerance, and help resolve inflammation. Upon completion of the degradation and phagocytosis of apoptotic cells, efferocytosis encourages macrophages to adopt a pro-resolution phenotype by decreasing the production of pro-inflammatory cytokines and increasing the levels of pro-resolving mediators. At the same time, it releases significant amounts of anti-inflammatory mediators, such as IL-10 (37) and TGF-β (38), as well as pro-resolving factors that help mitigate inflammation in IBD. These factors also stimulate the proliferation and differentiation of intestinal epithelial cells and fibroblasts, promoting healing of the intestinal mucosa in IBD (39). Besides these effects, efferocytosis also interacts with the immune system. For example, regulatory T cells have been shown to enhance efferocytosis in both in vitro and in vivo models (40). However, studies also indicate that TH17 cells play a critical role in the pathogenesis of IBD, with elevated numbers of Th17 cells present in the intestinal tissues of IBD patients (41). Infected apoptotic cells can induce Th17 differentiation, triggering intestinal inflammation. Pathogens often induce cell apoptosis, which may preferentially activate Th17-mediated immunity, contributing to IBD (42, 43). This suggests that while efferocytosis generally promotes anti-inflammatory and resolution responses, it may also lead to inflammation under specific conditions. Further research is required to better understand the connection between IBD and efferocytosis (**Figure 1**).

## 3 Multiple mechanisms restrict the development of IBD through efferocytosis

**Figure 2:**
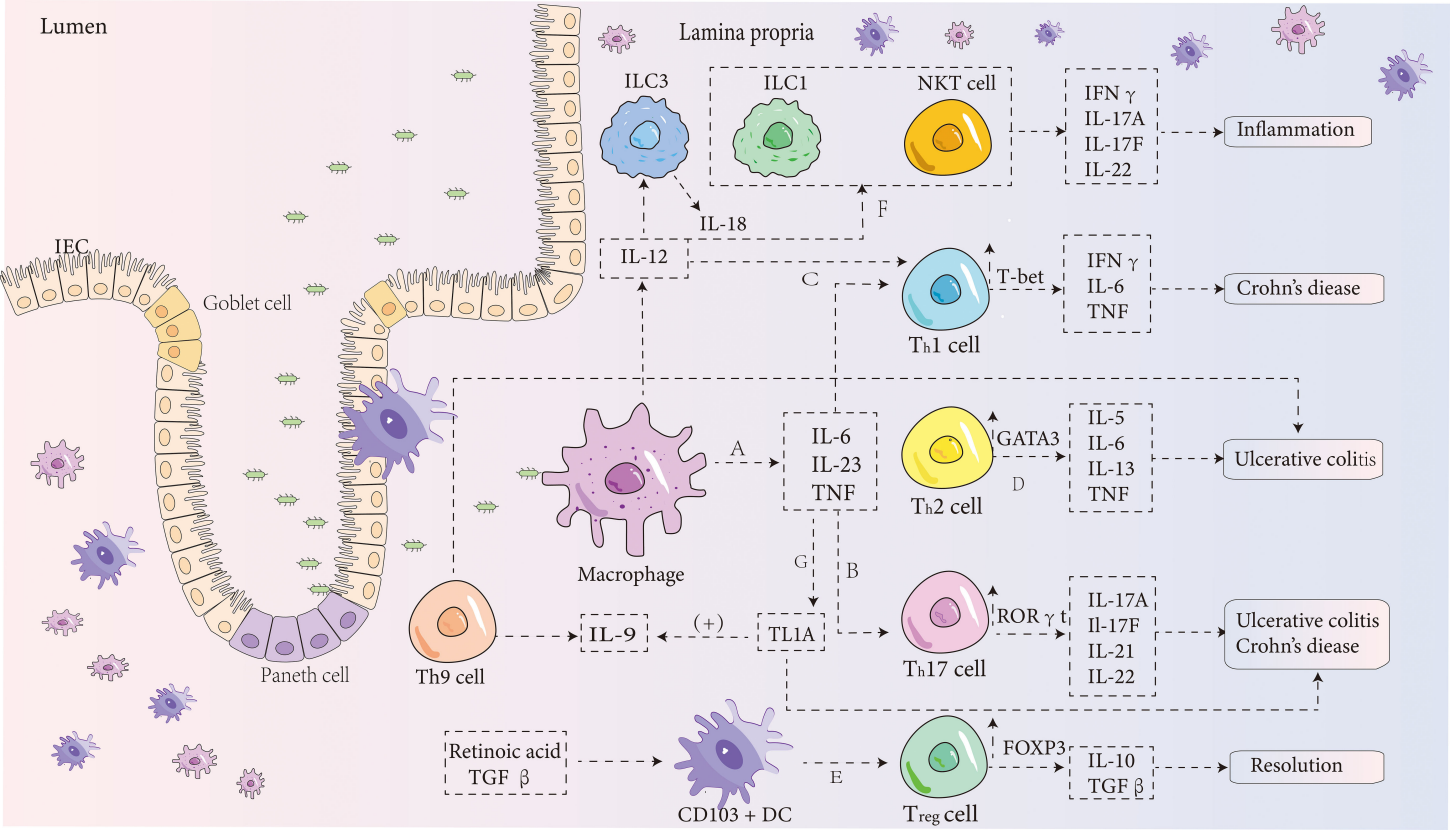
IBD pathogenesis diagram. **(A)** In the intestinal lamina propria, macrophages can stimulate the production of pro-inflammatory cytokines such as IL-6, IL-23, and TNF. IL-6 binds to its receptor in a soluble form, activating T cells and triggering the translocation of STAT-3. This process then promotes the transcription of anti-apoptotic genes such as Bcl-2 and Bcl-xl, which reduces T-cell apoptosis, thereby intensifying intestinal inflammation (54). **(B)** IL-6 also induces the differentiation of CD4+ T cells into Th17 cells (55), which release cytokines such as IL-17A, IL-17F (56), IL-21 (57), and IL-22. These cytokines, together with IL-23, contribute significantly to the development of IBD (56). During this process, the expression of the transcription factor RORγt in Th17 cells is enhanced. **(C)** Under the combined influence of IL-12 and IL-6, CD4+ T cells differentiate into Th1 cells (55), which mainly produce IFNγ, IL-6, and TNF, while increasing the expression of T-bet and STAT4. This leads to an exaggerated Th1 cell response, which is a major contributor to the development of Crohn’s disease. **(D)** Stimulation by IL-4 leads to the differentiation of CD4+ T cells into Th2 cells, which release cytokines such as IL-5, IL-6, IL-13, and TNF (58). Research has shown that NKT cells in ulcerative colitis release more IL-13, a Th2 cytokine, than T cells in Crohn’s disease (58). Thus, Crohn’s disease is typically associated with a Th1 response, while ulcerative colitis is thought to be driven by Th2 cells. However, this distinction is still debated. **(E)** Besides adaptive immune responses, innate immune mechanisms also play a role in IBD. CD103+ DCs, which depend on retinoic acid and TGF-β, promote the development of Foxp3+ Tregs, which release IL-10 and TGF-β (59). These cytokines have a suppressive effect on inflammation and protect against the development of IBD. **(F)** NKT cells, a subset of lymphocytes, can be activated indirectly by cytokines such as IL-12 and IL-18 secreted by innate lymphoid cell group 3 (60). These cells rapidly produce Th1, Th2, and Th17 cytokines, contributing to inflammation. **(G)** TNF is a key pro-inflammatory cytokine involved in IBD pathogenesis through several pathways. Recent studies indicate that TNF-like ligand 1A (TL1A) is a crucial mediator of intestinal inflammation (61). TL1A enhances the ability of IL-12, IL-4, or IL-23 to promote the differentiation of Th1, Th2, and Th17 cells (62, 63). It may also induce Th9 differentiation and increase IL-9 secretion by upregulating TGF-β and IL-4 expression, exacerbating DSS-induced colitis in mice (64).

IBD is an idiopathic condition primarily characterized by symptoms like rectal bleeding and weight loss, stemming from chronic and excessive inflammation of the gastrointestinal tract (44). IBD development involves a range of factors, both genetic and environmental. Genetic studies, including GWAS, have revealed more than 200 genetic loci associated with IBD (45), with the nucleotide-binding oligomerization domain 2 (NOD2) being one of the most prominent loci. Mutations in this locus can activate the NF-κB inflammatory pathway and initiate autophagy (46), and studies suggest that this factor can suppress macrophage function. Consequently, the accumulation of apoptotic cells that enhances efferocytosis might play a crucial role in terminating the inflammatory response (47). Moreover, the gut microbiota has been shown to contribute significantly to IBD pathogenesis, likely due to abnormal host-microbiota interactions affecting the immune system (48). Immune dysfunction in IBD is marked by epithelial damage and a failure to properly regulate immune responses (49), which include both innate and adaptive immune dysfunctions. This leads to abnormalities in intestinal epithelial cells (IECs), dendritic cells (DCs), macrophages, T cells, and other factors. In this process, several molecules such as TNF-α (50), IL-6 (49), IL-9 (51), IL-23 (52), and IL-17 (53) may be dysregulated, but efferocytosis can suppress these molecules while promoting the release of anti-inflammatory mediators (39). The following discussion will describe how efferocytosis can limit the progression of IBD from multiple angles (**Figure 2**).

### 3.1 Efferocytosis restricts IBD through multiple metabolic pathways

Macrophages are essential for maintaining intestinal immune homeostasis and have recently been identified as a potential target for treating inflammatory bowel disease (IBD). Several studies have highlighted the strong connection between macrophage activation and cellular metabolism (65). In the context of IBD, various immunometabolic alterations influence macrophage phenotype and function by regulating metabolic reprogramming and transcription, which in turn affects disease progression. Research indicates that during efferocytosis, a specific family of solute carrier (SLC) genes undergoes modification. At different stages of efferocytosis, various SLCs and other molecules are activated, facilitating SLC2A1-mediated aerobic glycolysis while suppressing oxidative phosphorylation. Furthermore, SLC16A1 contributes to the release of lactate, which promotes anti- inflammatory effects (18, 66). Through this process, SLC2A1-mediated glucose uptake and glycolytic intermediates enhance actin polymerization during efferocytosis, facilitating the uptake and clearance of apoptotic cells (18). Recent findings suggest that activation of 6-phosphofructo-2-kinase/fructose- 2,6-bisphosphatase 2 (PFKFB2) boosts glycolysis, increasing lactate release and regulating the expression of efferocytosis receptors like MerTK and LRP1, which extend the efferocytosis process (67). Thus, whether through SLC or PFKFB2-mediated glycolysis, there is a clear link between glycolysis and efferocytosis, although additional mechanisms remain to be explored.

The resolution of inflammation in efferocytosis is often closely tied to cellular metabolic processes. Studies have pointed to a unique relationship between apoptotic cell breakdown, fatty acid oxidation (FAO), and mitochondrial respiration during efferocytosis. One study observed that after efferocytosis, long-chain fatty acids accumulated in macrophages, activating the respiratory chain and generating metabolic intermediates that promoted macrophage anti-inflammatory responses (68). Although the direct link between this pathway and IBD was not identified, this non-classical mitochondrial response plays a crucial role in tissue repair and healing during damage and may offer new therapeutic avenues for IBD in the future. Other research suggests that efferocytosis can activate the tryptophan (TRP) pathway, triggering several pro-resolving programs, such as the induction of pro-resolving proteins, which further enhance efferocytosis (69). This study, using atherosclerosis as a model, connects the resolution of inflammation with efferocytosis and provides a theoretical basis for treating similar chronic inflammatory diseases like IBD. Additionally, macrophage arginase 1 (Arg1) and ornithine decarboxylase (ODC) convert apoptotic cell-derived arginine and ornithine into agmatine, thereby promoting efferocytosis (70). This research, using atherosclerosis as a model, demonstrates that disruption of this metabolic pathway can impair efferocytosis, leading to further inflammation and tissue necrosis in chronic inflammatory conditions. Conversely, enhancing this pathway could break the pathological cycle, offering new therapeutic possibilities for chronic inflammatory diseases.

### 3.2 Nuclear receptor gene deficiency in IBD, efferocytosis restricts IBD by activating nuclear receptors.

Defects in efferocytosis can lead to the development of various inflammatory and autoimmune disorders. Macrophage nuclear receptors, such as peroxisome proliferator-activated receptor (PPARγ) and liver X receptor (LXR), are involved in these processes. Research has shown that LXR expression is notably reduced in the intestinal tissues of IBD patients (71, 72). In vivo stimulation of apoptotic cells can activate LXR, leading to an increase in the expression of the apoptotic cell recognition receptor tyrosine kinase Mertk. This activation enhances efferocytosis, helping prevent and treat DSS-induced colitis and ulcerative colitis (71, 73). PPARγ and LXR have similar roles, and their mechanisms of action share notable similarities. Studies have suggested that activating PPARγ can effectively reduce the signs and symptoms of IBD and its progression (74). During macrophage efferocytosis, PPARγ functions as a transcriptional sensor of dying cells, responding to apoptotic cell signals and coordinating their clearance, which promotes self-tolerance and alleviates IBD (75). In this process, macrophage clearance of apoptotic cells reduces the production of reactive oxygen species, which are pro-inflammatory mediators activated by PPARγ (76). Additionally, PPARγ activation further enhances macrophage efferocytosis (77).

In the early phase of efferocytosis recognition, the externalization of intracellular phospholipids and extracellular molecules on apoptotic cells leads to the binding of proteins S and Gas6, activated by bridging molecules like TIM4, to phosphatidylserine (PS), and subsequently to the TAM family receptors. This binding activates downstream transcription factors such as LXRα, LXRβ, and PPARγ, which suppress inflammatory signaling pathways in macrophages (78, 79), thereby inhibiting the inflammatory response and preventing the continued development of IBD.

### 3.3 Efferocytosis engulfs neutrophil extracellular traps to restrict IBD

Neutrophils have a dual role in inflammation. During acute inflammation, they are rapidly recruited to the affected site to eliminate pathogens and mediate inflammatory responses through various pathways. In chronic inflammation, the role of neutrophils has also gained attention. Studies have shown that neutrophil extracellular traps (NETs) are present in the inflamed colon, where they can worsen tissue damage and contribute to thrombotic tendencies during active IBD (80). Additionally, NETs may disrupt the intestinal mucosal barrier through several mechanisms (81). NETs represent a distinctive form of cell death, occurring when dying neutrophils release their nuclear DNA, which combines with cytoplasmic proteins to form a unique structure (82). Efferocytosis can regulate the release of NETs by DNase I-dependent digestion, reducing their accumulation (83), which is significant in inhibiting the progression of IBD. Furthermore, research indicates that in autoimmune vasculitis (AAV), NETs accumulate, and blocking CD47 can prevent disease progression by restoring efferocytosis (84). Although this study did not directly examine this pathway in IBD, the similarities in the pathogenesis of AAV and IBD may offer valuable insights for IBD research. In addition to releasing NETs, apoptotic neutrophils also secrete reparative proteins, such as Annexin A1, which interacts with FPR2. This interaction can inhibit the recruitment of inflammatory cells by reducing integrin activation triggered by chemokines, while simultaneously enhancing macrophage efferocytosis (85, 86). Neutrophils also release antimicrobial peptides like α-defensins, which suppress macrophage mRNA translation, thereby increasing macrophage phagocytic activity and reducing the production of pro-inflammatory cytokines (87, 88), which helps to limit the development of IBD.

### 3.4 Efferocytosis restricts the development of IBD through immunological mechanisms

The complex pathogenesis of IBD results from the intricate interactions between the gut microbiota, environmental factors, and the immune system. Immunological mechanisms underlying IBD involve dysregulation of both innate and adaptive immunity. TH17 cells are critical in IBD development, as demonstrated by elevated levels of IL-17A and IL-17F in the inflamed intestinal mucosa of IBD patients (56). In addition to producing IL-17, TH17 cells also promote the secretion of other effector cytokines, including IL-9 and IL-22. These cytokines, along with TH9/IL-9, perpetuate a pro- inflammatory loop that contributes to IBD progression (89). Efferocytosis, the process of clearing apoptotic cells, plays a key role in the production of anti-inflammatory mediators such as TGF-β, PGE2, and IL-10. IL-25 has been shown to regulate TH1/TH17 immune responses in an IL-10- dependent manner (90), thereby slowing IBD progression. This highlights IL-25 as a promising candidate for therapeutic strategies in IBD treatment. Furthermore, Treg cells have been observed to enhance IL-13 secretion during the resolution of inflammation, which stimulates macrophages to release IL-10. The released IL-10 activates macrophage Vav1 through autocrine or paracrine signaling, resulting in Rac1 activation and promoting macrophage efferocytosis. This series of events aids in controlling inflammation and provides a potential new therapeutic approach for treating chronic inflammatory diseases (40).

### 3.5 Autophagy genes enhance efferocytosis to restrict the development of IBD

Autophagy plays a role in enhancing macrophage phagocytosis of apoptotic cells, and efferocytosis shares several regulatory factors with classical autophagy, particularly in terms of fusion and degradation with lysosomes. There is a strong association between deficiencies in autophagy genes and the enhancement of efferocytosis in various diseases (91). Research has shown that mice deficient in the autophagy-related gene NRBF2 experience impaired fusion of phagosomes containing apoptotic cells and lysosomes in macrophages through the MON1-CCZ1-Rab7 module. These mice are more susceptible to DSS-induced colitis, exhibiting severe intestinal inflammation and an accumulation of apoptotic cells (92). Thus, the lack of autophagy genes may lead to defective macrophage efferocytosis, contributing to the worsening of symptoms in several inflammatory diseases, including IBD.

### 3.6 Multiple mechanisms inhibit inflammatory responses through efferocytosis to restrict the development of IBD

Glucocorticoids (GCs) are frequently used to treat inflammatory conditions such as IBD. GCs activate the glucocorticoid receptor (GR), which suppresses pro-inflammatory genes while activating anti-inflammatory genes (93). The glucocorticoid-induced leucine zipper (GILZ), a well-know target of GR, mediates anti-inflammatory and pro-resolution effects, promoting macrophage reprogramming and enhancing efferocytosis. This reprogramming leads to the polarization of macrophages toward the M2 phenotype, initiating pro-resolution programs (94), which reduce inflammatory responses. IL-23 is also a significant pro-inflammatory cytokine in the pathogenesis of ulcerative colitis (UC) and Crohn’s disease (CD) (95). The uptake of apoptotic neutrophils by macrophages can reduce IL-23 transcription, thereby inhibiting inflammatory responses (96). Moreover, apoptotic cells heavily depend on TGF-β to coordinate anti-inflammatory responses in macrophages and to suppress the production of inflammatory mediators (97).

### 3.7 Various other cells in the intestinal tissue act as phagocytes to enhance efferocytosis and limit inflammatory responses in IBD

**Figure 3:**
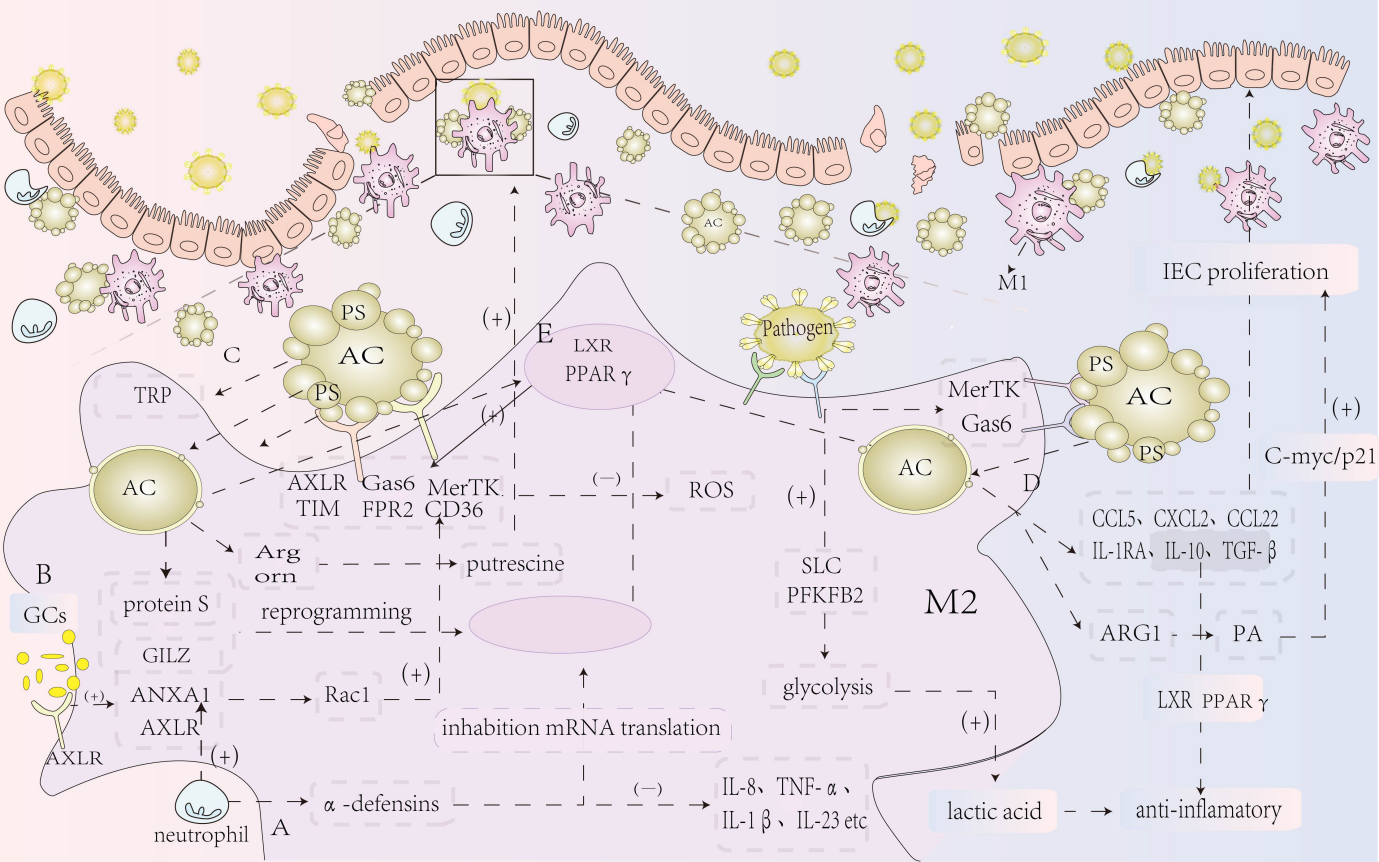
Summary of the mechanism of efferocytosis in IBD. **(A)** Neutrophils release antimicrobial peptides, such as α-defensins, which inhibit macrophage mRNA translation, thereby reducing the production of pro-inflammatory cytokines and lessening the inflammatory response. **(B)** Glucocorticoid treatment increases the expression of AnxA1 and its receptor ALXR, which regulate the inflammatory response, boost Rac1 activity in macrophages, and promote efferocytosis through CD36, reducing intestinal inflammation and maintaining homeostasis. Additionally, glucocorticoids elevate the expression of GILZ, a leucine zipper protein, which, together with protein S secreted by neutrophils, mediates anti-inflammatory and pro-resolution effects, enhancing macrophage reprogramming and efferocytosis. **(C)** Efferocytosis triggers the activation of the SLC gene family, promoting lactate release, which exerts anti-inflammatory effects. It also activates the tryptophan (TRP) pathway to promote key pro-resolution programs, including the induction of pro-resolving proteins, thereby enhancing efferocytosis. Moreover, macrophage arginase 1 (Arg1) and ornithine decarboxylase (ODC) convert apoptotic cell-derived arginine and ornithine into putrescine, further enhancing efferocytosis and reducing the inflammatory response. **(D)** Following efferocytosis, various chemokines (CCL5, CXCL2, CCL22) and anti-inflammatory mediators (IL-10 and TGF-β) are released, helping to suppress the inflammatory response and repair damaged intestinal mucosal epithelium. **(E)** Nuclear genes in macrophages regulate the transcription of surface receptors, enhancing efferocytosis and suppressing inflammation. Deficiencies in macrophage nuclear receptor genes may promote the development of IBD, while efferocytosis can activate nuclear receptors to prevent IBD development.

In the DSS-induced colitis model, BAI1-deficient mice display more severe colitis, characterized by an increased accumulation of unresolved apoptotic cells and higher levels of inflammatory cytokines. Targeted overexpression of BAI1 in colonic epithelial cells, which enhances efferocytosis, can significantly reduce colonic inflammation (98). Paneth cells, specialized epithelial cells in the intestine, function as phagocytes, facilitating the uptake of surrounding apoptotic cells to help maintain intestinal homeostasis (99). These cells play a crucial role in the progression of IBD. The autophagy-related gene ATG16L1 is a genetic risk factor that plays a key role in CD. Paneth cells with a deficiency in Atg16L1 exhibit significant abnormalities in the efferocytosis pathway and are closely linked to adipokines, leptin and adiponectin, which directly influence responses to intestinal damage, thus affecting the intestinal epithelium and contributing to the progression of CD (100, 101). Consequently, promoting efferocytosis in Paneth cells and intestinal epithelial cells may effectively reduce intestinal inflammation in patients with mucosal inflammation, offering potential therapeutic implications for IBD and other inflammatory bowel diseases (**Figure 3**).

## 4 Targeting efferocytosis in IBD treatment

IBD is a prevalent inflammatory condition affecting individuals globally, with various treatment options available. Traditionally, IBD has been managed through drug therapies aimed at controlling symptoms, such as aminosalicylates (102), corticosteroids (CS) (103), immunomodulators, and biologics (104, 105), with surgical resection and other general measures utilized as needed. In recent years, new treatment strategies have emerged to improve clinical outcomes, including apheresis therapy (106), modulation of gut microbiota (107), and cellular therapies (108). Besides Western medical treatments, traditional Chinese medicine (TCM) offers distinct advantages in treating IBD. For example, baicalin has been shown to increase interferon factor 4 protein expression and reverse LPS-induced macrophage activation, thereby modulating macrophage polarization and enhancing inflammatory responses in DSS-induced colitis in mice (109). Indirubin, a key component of Qingdai, has been found to alleviate DSS-induced colitis in mice when administered orally (110). However, research on how TCM regulates the immune microenvironment remains insufficient, with a primary focus on anti-inflammatory effects and a lack of novel approaches. As treatment strategies continue to advance, the focus of IBD management has shifted from simply addressing clinical symptoms and preventing complications to promoting healing of the colonic mucosa, evidenced by the resolution of endoscopic ulcers (111, 112). Additionally, new and emerging treatment approaches continue to offer promising avenues for IBD therapy.

### 4.1 Single infusion of apoptotic cell therapy

Research suggests that a single infusion of apoptotic cells can significantly improve the clinical symptoms of DSS-induced colitis (47). Apoptotic cells can inhibit pro-inflammatory signaling pathways and serve as a primary mechanism for resolving inflammation, mainly through their suppression of the transcription factor NF-κB and inflammatory bodies. NF-κB plays a central role in regulating gene expression during inflammation, particularly in the production and secretion of IL-1β (113). In DSS-induced colitis models, treatments such as anti-IL-1β therapy and the inhibition of inflammatory bodies (114, 115) have shown that NF-κB can reduce the activity of macrophages and dendritic cells in the lamina propria, along with inflammatory bodies. Therefore, the accumulation of apoptotic cells at the inflammation site is likely a key mechanism in halting the inflammatory response. The development of IBD is influenced by both genetic and environmental factors, with DSS exposure contributing to disease onset. Additionally, studies have demonstrated that extracellular vesicles from apoptotic cells can promote TGF-β production in macrophages and suppress inflammatory responses in experimental colitis models (116). Consequently, a single infusion of apoptotic cells to trigger the resolution phase of inflammation via efferocytosis presents a promising treatment approach for IBD, marking a new area of exploration. However, there remains a lack of extensive research and practical experience in this field. Overall, it holds considerable potential for future IBD therapies.

### 4.2 Apoptotic cell clearance enhancers

Efferocytosis is a continuous process in the body, where the phagocytic capacity is both expansive and constrained. Studies have demonstrated that enhancing efferocytosis in intestinal epithelial cells and Paneth cells can improve the clearance of apoptotic cells and mitigate inflammatory responses (98, 99). In severe IBD cases, intestinal tissues often show an elevated number of apoptotic colonic epithelial cells (117, 118). This suggests that an increase in apoptotic cells or a malfunction in their clearance may contribute to the intensification of inflammation. Therefore, boosting efferocytosis in the early phases of IBD, accelerating the removal of apoptotic cells, and promoting efferocytosis by intestinal epithelial cells could be crucial in reducing inflammation later in the disease. Recent advancements have introduced a “chimeric efferocytosis receptor” that enhances macrophage efferocytosis, allowing them to engulf more apoptotic cells and initiate anti-inflammatory signaling, alleviating colitis symptoms. However, the findings are still preliminary, and the effectiveness is restricted to BAI1 and TIM-4 receptors, which have specific limitations. It remains uncertain whether this approach will function effectively in human inflammatory conditions, but it provides a new avenue for IBD treatment (119). In conclusion, from a pharmacological perspective, developing agents to enhance apoptotic cell clearance, including strategies that boost efferocytosis by intestinal epithelial cells and Paneth cells, represents a promising therapeutic approach for IBD that deserves further investigation.

### 4.3 Introducing pro-resolving factors for the treatment of IBD

As mentioned earlier, the pro-resolving factors secreted following efferocytosis can stimulate the proliferation and differentiation of various intestinal cells, aiding in the repair of the intestinal mucosa, while also releasing anti-inflammatory mediators to help resolve inflammation. These macrophage-secreted pro-resolving factors are collectively known as SuperMApo, a novel biological complex that includes all cytokines released by macrophages during efferocytosis, such as TGF-β, IL-10, IL-1RA, and others. These factors primarily coordinate the actions of fibroblasts and intestinal epithelial cells (IECs) to support mucosal healing and pro-resolution responses (120–122). This has been confirmed through three experimental models: the initial T-cell transfer model (123), the DSS-induced colitis model, and the biopsy forceps injury model of the colonic mucosa (124). From a resolution pharmacology perspective, introducing these pro-resolving factors in the early stages of IBD can enhance the clearance of apoptotic cells by IECs, thereby reducing inflammation in the later stages of the disease. Additionally, this process helps trigger mechanisms involved in mucosal healing (39) and promotes myofibroblast contraction to facilitate wound closure (125), making it a promising potential treatment approach for IBD (39).

### 4.4 Synthetic biomimetic drugs targeting efferocytosis for the treatment of IBD

Macrophages play a multifaceted role in the immune system and inflammatory responses, making them an attractive target for the treatment of various inflammatory diseases. Inspired by macrophages’ ability to selectively engulf apoptotic cells while leaving healthy cells unharmed, Zheng Ying and colleagues created a biomimetic drug delivery system (Effero-RLP) by hybridizing apoptotic red blood cell membranes with liposomes. This system targets inflammation via macrophage-mediated efferocytosis. The researchers encapsulated rosiglitazone (ROSI), an agonist of the anti-inflammatory peroxisome proliferator-activated receptor γ (PPAR-γ), within the delivery system. Through efferocytosis, ROSI is directed to the site of inflammation. In a DSS-induced colitis model, Effero-RLP significantly enhanced macrophage efferocytosis, induced polarization of macrophages to an anti-inflammatory phenotype, and accumulated in inflamed colonic tissues to treat IBD (126). Additionally, β-cyclodextrin, a biomimetic nanodrug sensitive to reactive oxygen species and coated with macrophage membranes, has shown high efficiency in treating UC (127). These biomimetic drug therapies offer several benefits, including enhanced clinical efficacy for IBD and precise drug delivery targeting. However, challenges such as material preparation and binding efficiency remain, and the approach still faces several hurdles before reaching clinical trials.

### 4.5 Enhancing efferocytosis directly with pharmacology for the treatment of IBD

COL is a biased agonist that partially occupies the ligand-binding pocket of formyl peptide receptor 2 (FPR2), a receptor involved in regulating inflammation. COL enhances efferocytosis by directly binding to FPR2 and has shown significant therapeutic effects in DSS-induced colitis (38). Additionally, macrophage efferocytosis receptors Axl and MerTK, which are IL-4-dependent, play a role in repairing damaged intestinal tissue, suggesting that targeting Axl/MerTK on macrophages could offer a new therapeutic approach for IBD treatment (128). These findings suggest that enhancing macrophage efferocytosis pharmacologically can alleviate colitis symptoms, and IBD is a disease that could benefit from such treatment. Therefore, this research provides valuable insights for developing new therapies for various IBD conditions.

## 5 Discussion

In recent years, an increasing number of studies have highlighted the connection between efferocytosis and IBD. The role of efferocytosis in IBD primarily involves two aspects: promoting pro-resolving responses and supporting the repair of the intestinal mucosa. This article outlines the efferocytosis process and its associated molecules, and reviews the key mechanisms by which efferocytosis affects IBD. These mechanisms include the secretion of pro-resolving factors that encourage the proliferation and differentiation of fibroblasts and intestinal epithelial cells (IECs) to repair the damaged mucosa (39), as well as the release of anti-inflammatory mediators such as TGF-β (38) and IL-10 (37), which help suppress intestinal inflammation. Furthermore, macrophages and other cells enhance efferocytosis by modifying metabolism and activating receptors, indicating the potential and feasibility of boosting efferocytosis to treat diseases like IBD. During the literature review, it was also found that intestinal epithelial cells and Paneth cells are involved in efferocytosis (98, 99), affecting the progression of IBD. Additionally, the immunological mechanisms underlying IBD are related to efferocytosis (41, 42). However, the current research still lacks direct studies linking IBD mechanisms with efferocytosis, with most studies focusing on inflammatory and pro-resolving responses. There is a need for more research exploring the relationship between IBD-associated immune mechanisms and efferocytosis.

A considerable body of evidence demonstrates a strong association between defects in macrophage efferocytosis and the progression of IBD (129), indicating the potential of macrophages as a novel therapeutic target for IBD. The present review examines various potential therapies targeting macrophage efferocytosis for IBD treatment, though most of these approaches lack practical application and corresponding advancements in drug research and development. Furthermore, questions regarding the long-term efficacy and real-world feasibility of these treatment methods remain unresolved, necessitating further in-depth and comprehensive investigations.

Research also emphasizes the link between IBD and the onset of colon cancer and other malignancies, with factors such as genetic predisposition, impaired intestinal barrier function, and immunological dysregulation underlying persistent intestinal inflammation in IBD (130). Both deficiencies and enhancements in efferocytosis have been implicated in promoting cancer progression (26). The defining features of UC and CD include chronic, unresolved gastrointestinal inflammation, which over time may lead to complications such as fibrosis, organ damage, and subsequent organ failure, including CD-associated strictures, abscesses, and fistula formation (131). Consequently, achieving a balance in the transition between M1 and M2 macrophages and optimizing the regulation of efferocytosis remains a critical area for further investigation, with significant implications for improving the clinical management of IBD patients.
